# Supplementary material for: Integrated Transcriptome and Metabolome Analyses Reveal Details of the Molecular Regulation of Resistance to Stem Nematode in Sweet Potato
Source: Plants (Basel). 2023 May 22;12(10):2052. doi: 10.3390/plants12102052 (PMC10221022; doi:10.3390/plants12102052)
Supplement: Supplementary file 1 [file plants-12-02052-s001.zip › Supplementary Figure legends.pdf]

Supplementary Figure legends

Supplementary Fig. S1. Number of differentially expressed genes (DEGs) between uninfected (control, CK) and infected sweet potato. (A) Number of DEGs at various time points in the resistant variety Zhenghong 22. (B) Number of DEGs at various time points in the susceptible variety Longshu 9.

Supplementary Fig. S2. Trend analysis of transcriptome data and KEGG analysis of selected modules. (A) Different modules were obtained by trend analysis of DEGs in the two varieties. (B) KEGG analysis of genes in 10 selected modules.

Supplementary Fig. S3. Transcriptome analysis with criterion adjusted to 10-times difference in transcript levels between pairs of samples, and KEGG analysis of DEGs. (A) Number of DEGs showing 10-times differences in transcript levels in the two sweet potato varieties at various time points. (B) KEGG analysis of DEGs.

Supplementary Fig. S4. PCA based on transcriptome data. (A) PCA diagram based on metabolites detected in negative ion mode. (B) PCA diagram based on metabolites detected in positive ion mode.

Supplementary Fig. S5. Trend analysis of metabolome data and KEGG analysis of selected modules. (A) Different modules obtained by trend analysis of DMs between the two varieties. (B) KEGG analysis of metabolites in four selected modules.

Supplementary Fig. S6. Analysis of DMs showing at least 10 times difference in abundance between pairs of samples. (A) Number of metabolites showing at least 10 times difference between pairs of samples. (B) KEGG analysis of DMs.

Supplementary Fig. S7. Selection of modules related to resistance to stem nematode from WGCNA.

Supplementary Fig. S8. Correlation heatmap of the top 100 DEGs and top 100 DMs.

Supplementary Fig. S9. Genes and metabolites responding to stem nematode infection in the resistant cultivar Zhenghong 22. (A) Genes in secondary metabolite synthesis pathway. (B) Genes in MAPK signaling pathway-plant. (C) Genes in plant hormone signaling transduction pathway. (D) Genes in plant-pathogen interaction pathway. (E) Metabolites accumulated after stem nematode infection.
