# Supplementary material for: Integrated Transcriptome and Metabolome Analyses Reveal Details of the Molecular Regulation of Resistance to Stem Nematode in Sweet Potato
Source: Plants (Basel). 2023 May 22;12(10):2052. doi: 10.3390/plants12102052 (PMC10221022; doi:10.3390/plants12102052)
Supplement: Supplementary file 1 [file plants-12-02052-s001.zip › Supplementary Table S1 and Supplementary Table S2.pdf]

Supplementary Table S1. Quantitative Real-time PCR primer design

Genes	L-primer	R-primer
itf10g20620.t1	TCACTTGGTCTTGGGAAACC	GCAGAAGACCTGCATCACAA
itf04g08530.t1	GAGGGAGATGTGATGGGAGA	CCACCCCTTCCTTAATTTCC
itf14g18430.t1	AAACCAGATGGCAATGGAAG	TCAGCAACACTGCAAGCTCT
itf14g17940.t1	GCCACCTTGTTTCTTTGCAT	TCTCAGAGACAGCAGCTCCA
itf06g12490.t1	ATTGGTTCAGGCAGTTTTGG	GCCTTAAAGTCATCGGCAAA
itf10g03250.t1	GTTGCAGGTCCCTTCCATTA	TTTCTTGGGGCATTTGAAAG
itf12g18840.t1	ATGAAGTCGATGAGACAGACACA	ATCTGGATAACACCCTGAGGATT
Actin	AGCAGCATGAAGATTAAGGTTGTAGCAC	TGGAAAATTAGAAGCACTTCCTGTGAAC

Supplementary Table S2. Annotation of metabolites in correlation network diagram (Fig. 6).

Index	Name	Index	Name
M84T352_POS	1-Aminocyclopropanecarboxylic acid	M159T326_NEG	2-Oxoadipic acid
M291T426_POS	Argininosuccinic acid	M173T436_NEG	Cis-aconitate
M277T54_POS	cis-9-Palmitoleic acid	M129T437_2_NEG	Citraconic acid
M131T220_POS	Coniferyl alcohol	M115T200_NEG	Fumarate
M539T68_POS	Cucurbitacin d	M583T161_NEG	Gambogic acid
M228T166_POS	Deoxycytidine	M116T299_NEG	Indole
M375T496_POS	Glu-Ala-Arg	M129T61_NEG	Ketoleucine
M613T495_POS	Glutathione, oxidized	M279T37_NEG	Linoleic acid
M117T333_POS	Succinamide	M147T257_NEG	Mevalonic acid
M149T221_POS	trans-Cinnamate	M433T175_NEG	Naringenin-7-o-glucoside
M352T99_POS	trans-Zeatin-riboside	M88T343_NEG	Sarcosine
M338T355_2_POS	Tyr-Arg	M187T263_NEG	Val-Ala
M120T247_POS	Tyramine	M302T193_NEG	Val-Trp
M599T246_NEG	1-Octadecanoyl-sn-glycero-3-phospho-(1'-myo-inositol)		
