# Supplementary figures and images for: Integrated Transcriptome and Metabolome Analyses Reveal Details of the Molecular Regulation of Resistance to Stem Nematode in Sweet Potato
Source: Plants (Basel). 2023 May 22;12(10):2052. doi: 10.3390/plants12102052 (PMC10221022; doi:10.3390/plants12102052)

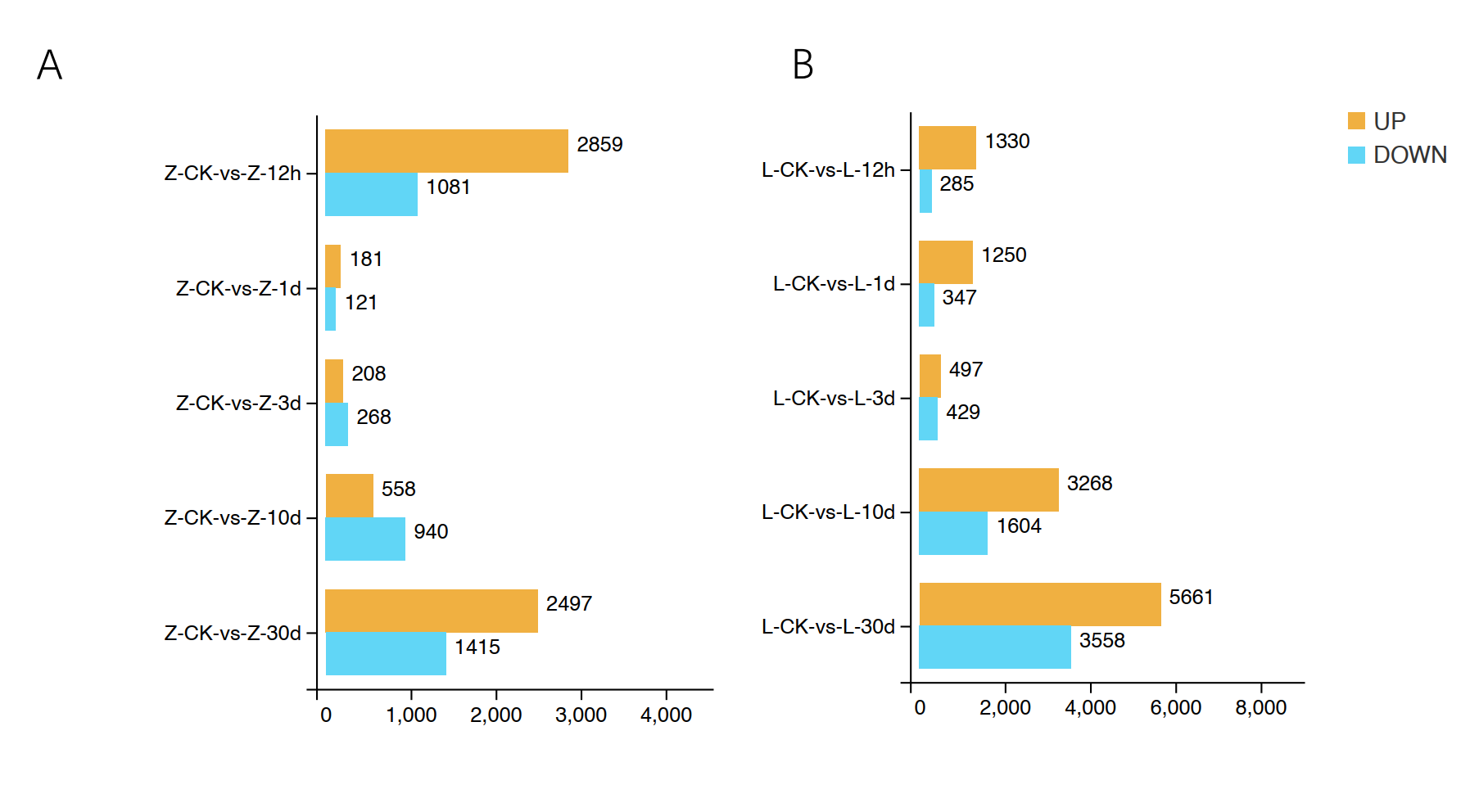

Supplement: Supplementary file 1 [file plants-12-02052-s001.zip › Supplementary Fig/Supplementary Fig. S1.png]

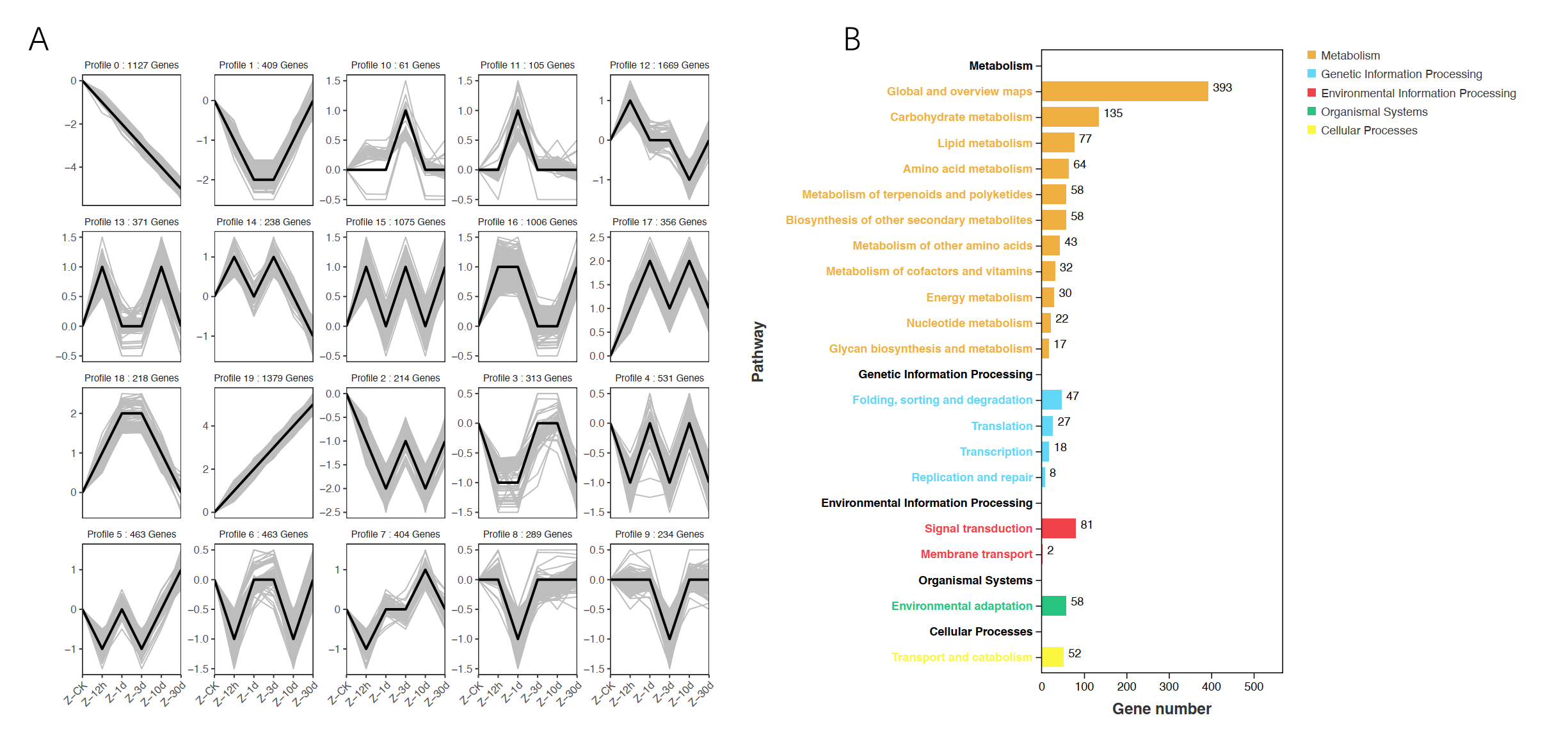

Supplement: Supplementary file 1 [file plants-12-02052-s001.zip › Supplementary Fig/Supplementary Fig. S2.png]

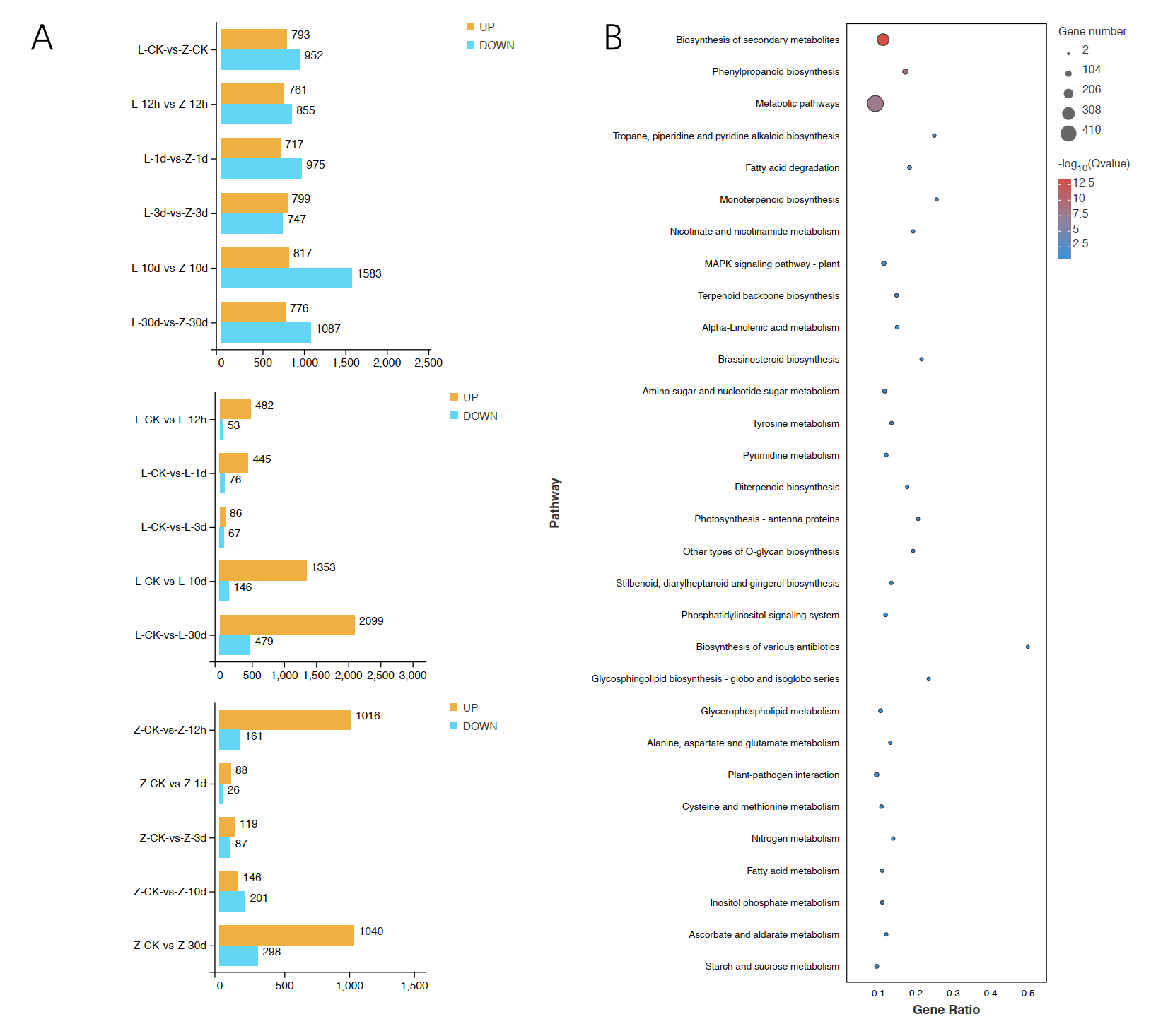

Supplement: Supplementary file 1 [file plants-12-02052-s001.zip › Supplementary Fig/Supplementary Fig. S3.png]

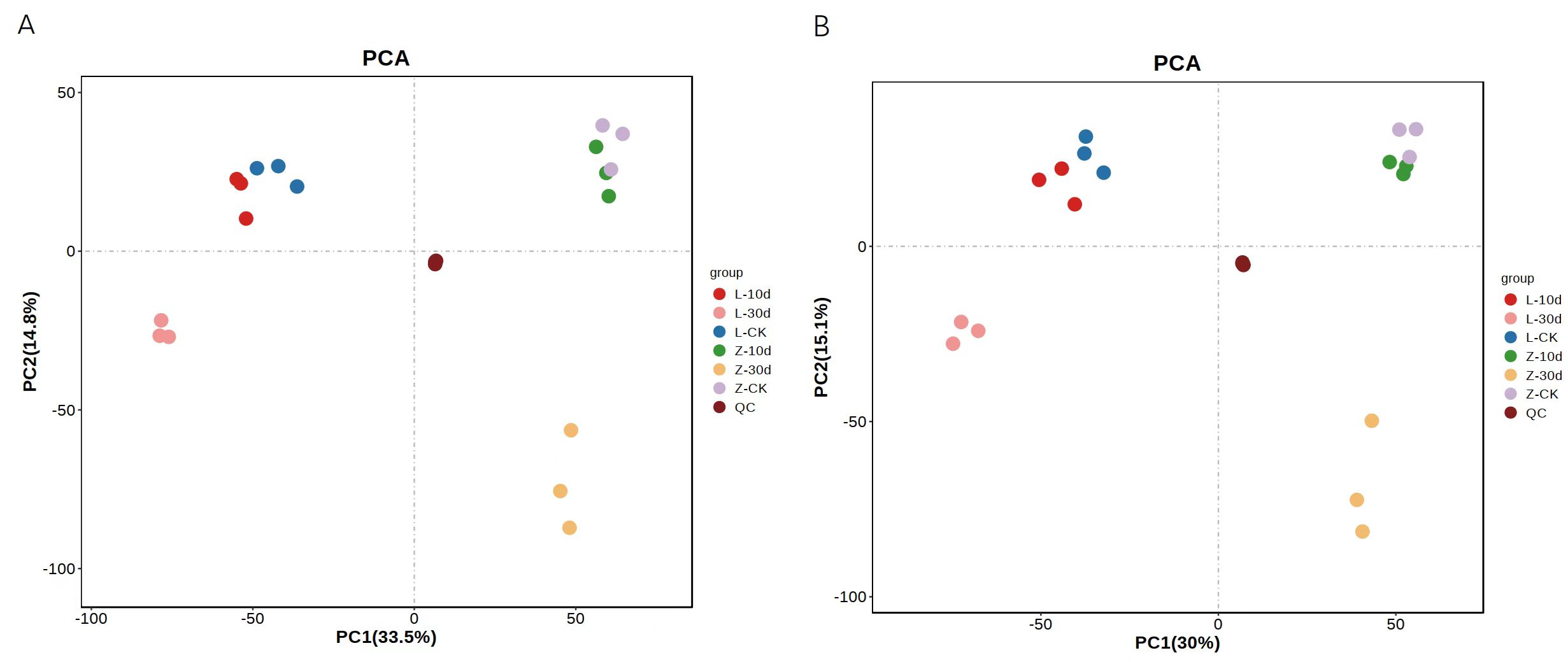

Supplement: Supplementary file 1 [file plants-12-02052-s001.zip › Supplementary Fig/Supplementary Fig. S4.jpg]

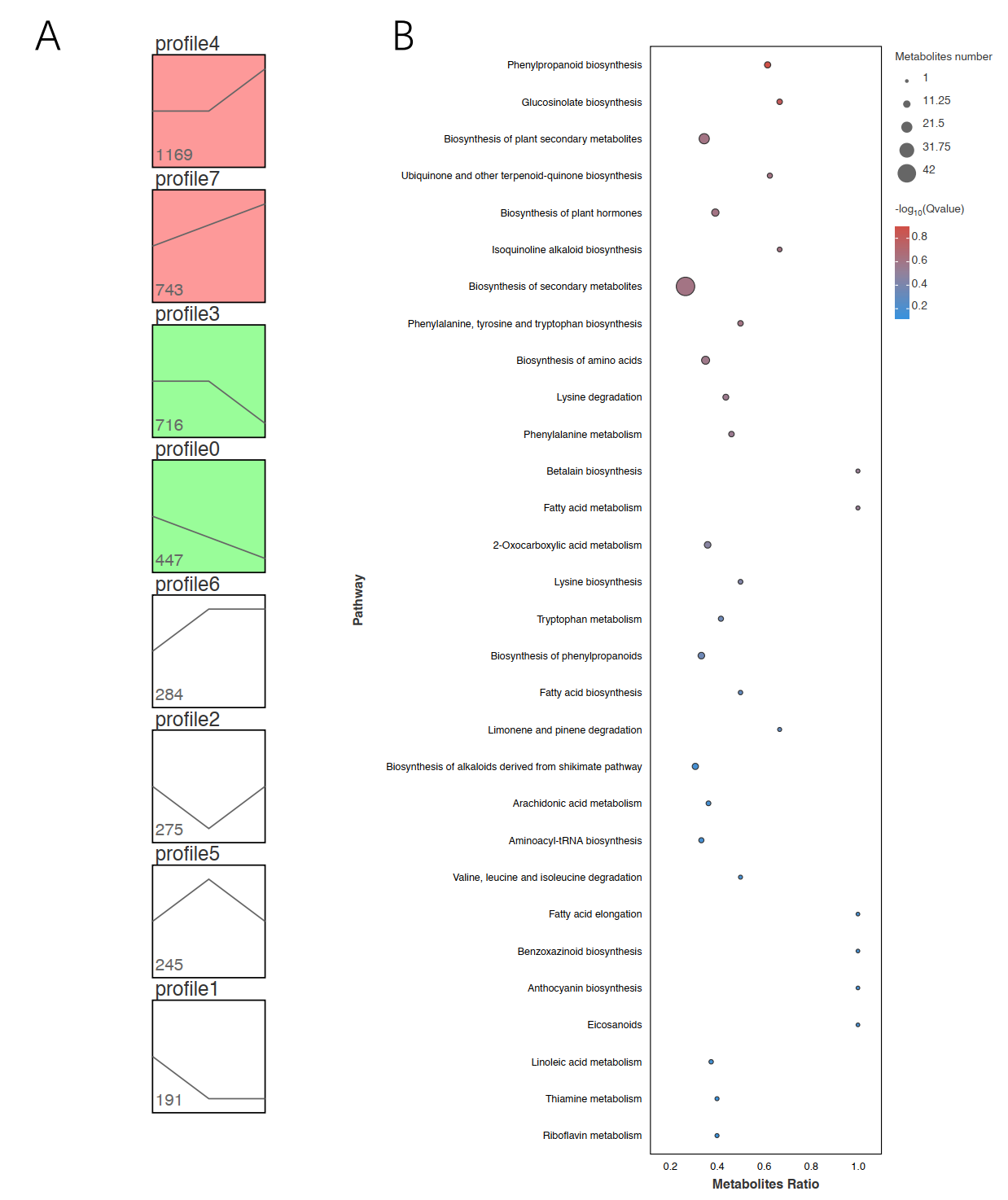

Supplement: Supplementary file 1 [file plants-12-02052-s001.zip › Supplementary Fig/Supplementary Fig. S5.png]

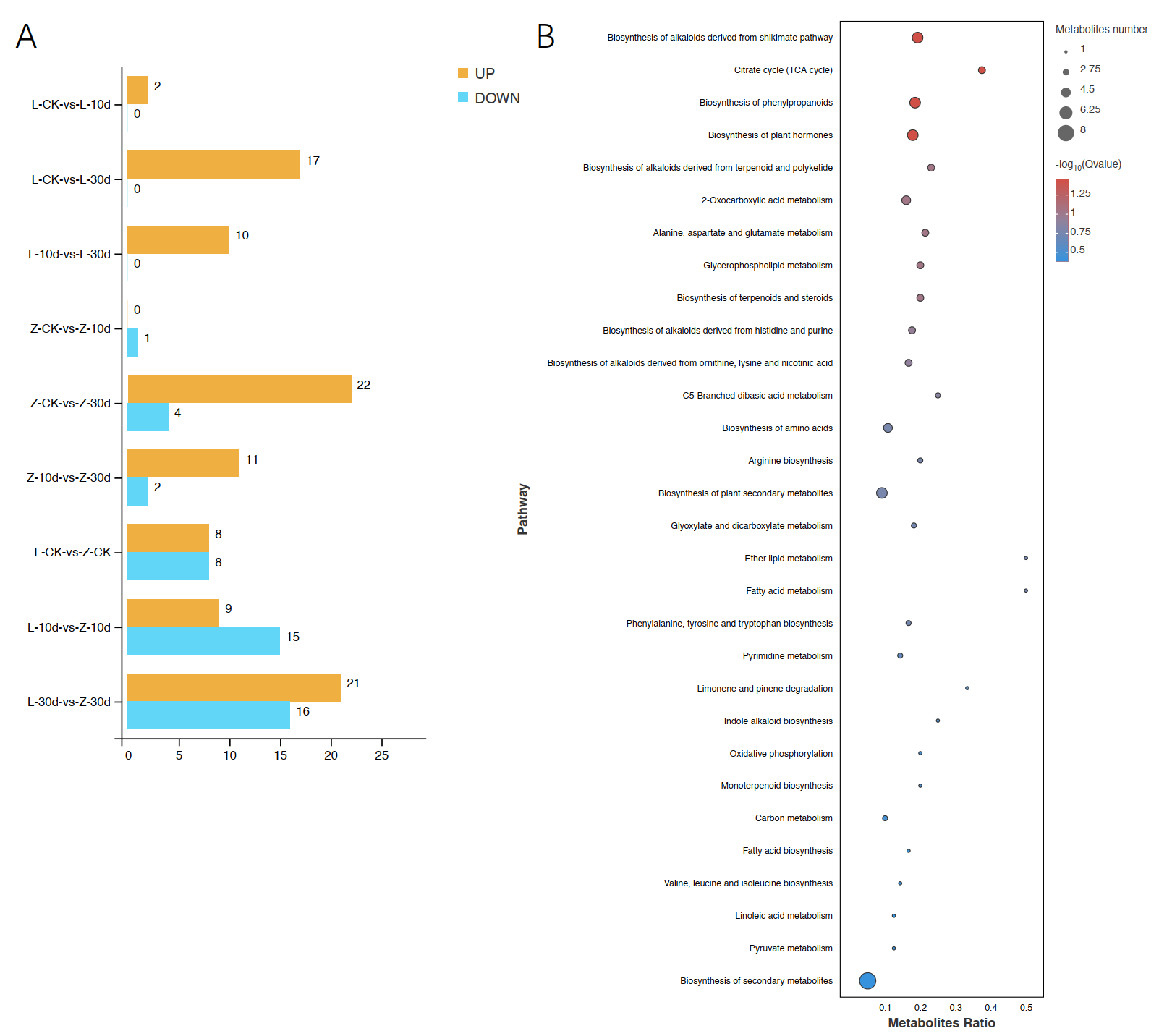

Supplement: Supplementary file 1 [file plants-12-02052-s001.zip › Supplementary Fig/Supplementary Fig. S6.png]

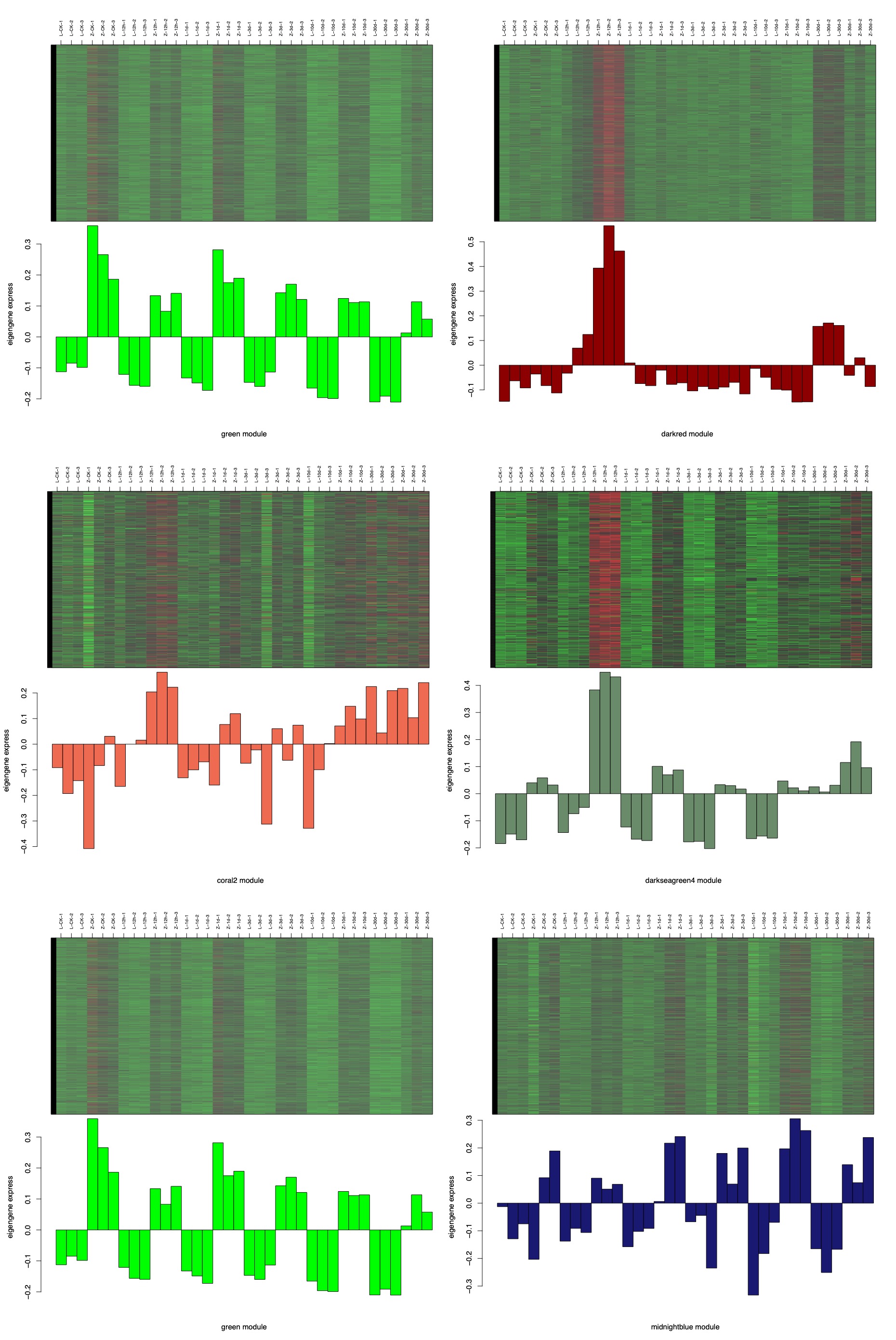

Supplement: Supplementary file 1 [file plants-12-02052-s001.zip › Supplementary Fig/Supplementary Fig. S7.jpg]

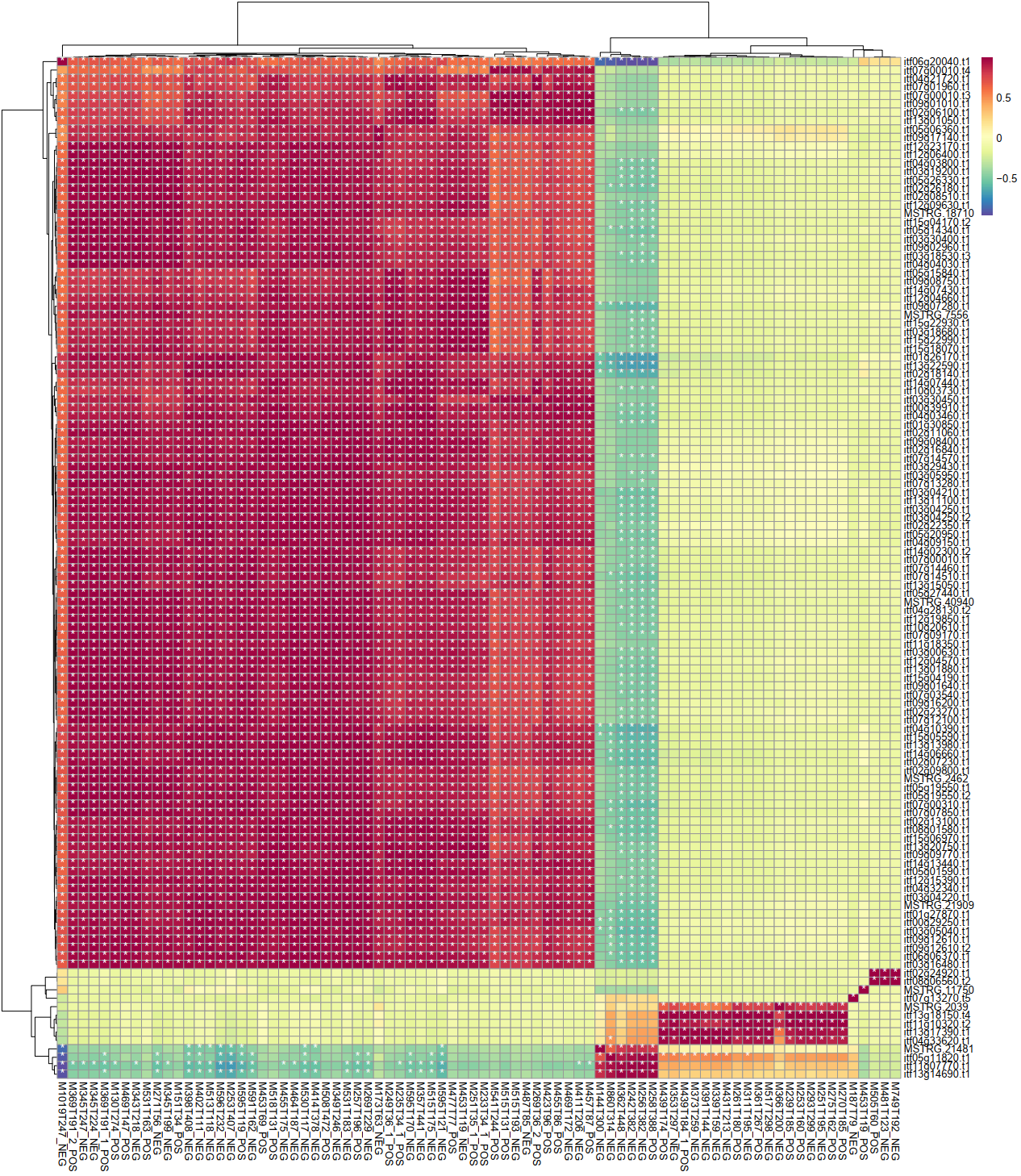

Supplement: Supplementary file 1 [file plants-12-02052-s001.zip › Supplementary Fig/Supplementary Fig. S8.png]

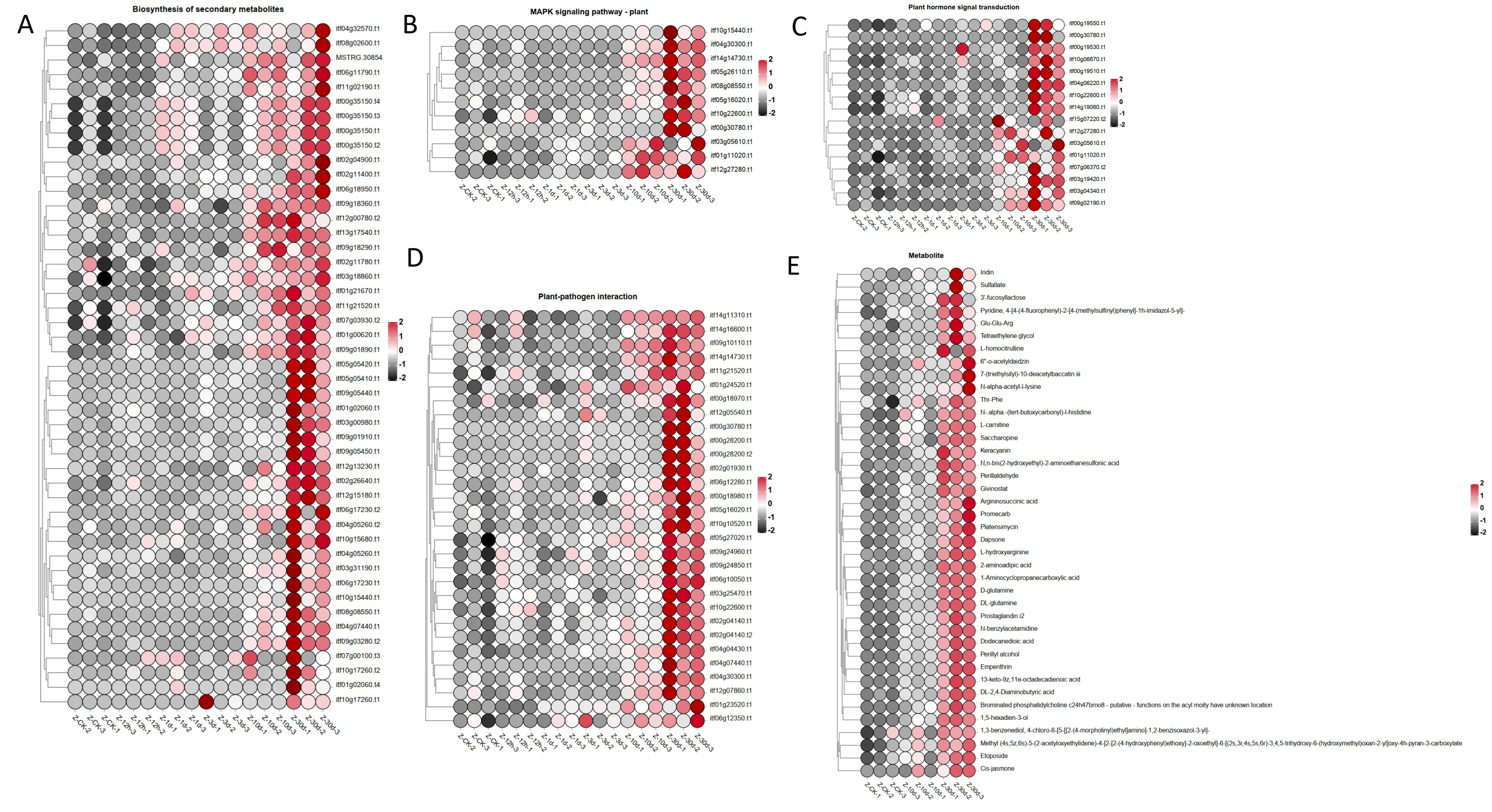

Supplement: Supplementary file 1 [file plants-12-02052-s001.zip › Supplementary Fig/Supplementary Fig. S9.png]
